# The final ecosystem goods and services Voltron: the power of tools together

**DOI:** 10.3389/fevo.2023.1290662

**Published:** 2023-12-21

**Authors:** Leah M. Sharpe, Matthew C. Harwell, Colin Phifer, George Gardner, Tammy Newcomer-Johnson

**Affiliations:** 1 Gulf Ecosystem Measurement and Modeling Division, Office of Research and Development, United States Environmental Protection Agency, Gulf Breeze, FL, United States; 2 Pacific Ecological Systems Division, Office of Research and Development, United States Environmental Protection Agency, Newport, OR, United States; 3 Science Department, Lane Community College, Eugene, OR, United States; 4 Performance Risk and Social Sciences Office, Social Science and Performance Division, Office of the Chief Financial Officer, National Oceanic and Atmospheric Administration, Silver Spring, MD, United States; 5 Watershed and Ecosystem Characterization Division, Office of Research and Development, U.S. Environmental Protection Agency, Cincinnati, OH, United States

**Keywords:** decision support tools, community decision-making, stakeholder engagement, ecosystem services, final ecosystem goods and services

## Abstract

Environmental decision-making benefits from considering ecosystem services to ensure that aspects of the environment that people rely upon are fully evaluated. By focusing consideration of ecosystem services on final ecosystem goods and services (FEGS), the aspects of the environment directly enjoyed, used, or consumed by humans, these analyses can be more streamlined and effective. The U.S. Environmental Protection Agency has developed a set of tools to facilitate this consideration. The central feature of FEGS is that ecosystems are viewed through the diverse ways people directly benefit from them. The National Ecosystem Services Classification System (NESCS) Plus provides a framework for describing and identifying FEGS consistently. The standardization made available by NESCS Plus allows other tools and databases to interact using the NESCS Plus architecture and taxonomy, providing diverse insights for decision makers. Here, we examine the synergy of using the following four tools together: (1) the FEGS Scoping Tool; (2) the FEGS Metrics Report; (3) the EnviroAtlas; and (4) the EcoService Models Library. The FEGS Scoping Tool helps users determine what ecosystem services are relevant to a decision by harnessing FEGS understanding to enable communities to identify the relative importance of beneficiaries relevant to a decision and biophysical aspects of the environment of direct relevance to those beneficiaries. The FEGS Metrics Report can guide which metrics to monitor or model to represent those priority services. The EnviroAtlas, a powerful tool containing geospatial data and other resources related to ecosystem services, chemical and non-chemical stressors, and human health, and the EcoService Models Library, a database of ecosystem models, are two tools that support users in mapping and modeling endpoints relevant to priority services. While each of these tools is valuable on its own, together, they provide a powerful approach to easily incorporate and operationalize ecosystem services efforts into different parts of decision-making processes across different types of decisions. We illustrate how these integrated tools can be used together with a hypothetical example of a complex environmental management case study and the combined benefit of using the FEGS tools together.

## 1 Introduction: ecosystem goods and services and final ecosystem goods and services

Humans are inextricably connected with the ecosystems in which we exist. Costanza et al. (1997) and the 2005 Millennium Ecosystem Assessment attempted to make this connection tangible through the concept of ecosystem goods and services (EGS)^1^, defined as the ecological characteristics, functions, or processes that directly or indirectly contribute to human well-being. Acknowledging these connections in decision-making is critical for ensuring that the aspects of the environment that relate to the decision are comprehensively considered. This can be challenging, however, as connections between ecosystems and humans are complex, nonlinear, and dynamic (Costanza et al., 2017). First, there may be synergies or tradeoffs among services. Second, contributions to well-being vary spatially and temporally. For example, people who live in cities enjoy EGS that can be influenced by ecosystems far away. Third, the magnitude of societal welfare effects depends on interactions between the ecosystem services provided and manufactured, human, and social capital. For example, the recreational and aesthetic benefits of a park, lake, or beach depend on their accessibility through walkways, boat ramps, etc. These complexities are further amplified by difficulties communicating these connections within and outside scientific communities and environmental decision-making bodies.

To improve communications with these audiences and linkages with social analyses to make the concept of EGS more actionable for decision makers, the concept of final ecosystem goods and services (FEGS) was developed. Final EGS are defined as those “components of nature, *directly* enjoyed, consumed, or used to yield human wellbeing” (Boyd and Banzhaf, 2007). As such, they represent only the subset of EGS that contribute to well-being (note: understanding indirect or intermediate EGS underlying FEGS is crucial if we wish to understand, assess, or manage them). FEGS are identified by focusing on the distinct ways in which humans benefit from the environment. Analyses using this approach view ecosystems as systems of production in which biophysical features and conditions are combined to produce socially valuable environmental outcomes and are explicit about the myriad ways humans benefit from those outcomes. These two aspects of FEGS, explicit acknowledgment of *how* humans benefit and *what* they are benefiting from, are critical as they support well-defined connections between different aspects of the ecosystem and specific beneficial uses that they are supporting. Because of these connections, using a FEGS approach can be a particularly effective way of incorporating EGS into decision-making (DeWitt et al., 2020).

Over the years, many decision support tools have been developed to incorporate EGS into decision-making (Harwell et al., 2023; Newcomer-Johnson et al., in press). These tools can be used for different types of decisions and at different points in the decision-making process. All of these tools support decision makers in various ways but they discuss EGS differently, which means that using them in concert requires decision makers to make decisions about how the various definitions of EGS connect to one another. These tools are developed for decision makers with limited time and resources, the additional time required to learn multiple EGS classification systems and to develop crosswalks amongst them can be an obstacle to tool adoption. The U.S. Environmental Protection Agency (EPA) has developed a set of tools that address different decision maker needs and can be used at different points in the decision process but use the same EGS framework. The burden of tool adoption and the obstacles to tool accessibility can be reduced through the use of a consistent and comprehensive EGS framework that supports integration of these tools and facilitated the incorporation of multiple tools into a decision-making process. Here, we review the advantages and disadvantages of common EGS classification systems for the purpose of demonstrating the aspects of the National Ecosystem Services Classification System (NESCS) Plus that made it particularly useful for decision-making and for EPA tool developers. We describe the EPA tools using the NESCS Plus framework and the synergistic potential arising from tool integration. Finally, we also suggest that the utility of these tools will continue to increase as new tools use and existing tools adopt this same EGS framework.

## 2 Classification systems

The complex connections between environmental changes and human well-being can make deliberate and focused consideration of ecosystem services challenging. Defining, measuring, quantifying, valuing, or accounting for EGS requires a collaborative effort among natural scientists and social scientists as environmental processes and functions produce potential EGS, and people, groups, or organizations enjoy, use, or consume EGS. This means that descriptions of EGS must be clearly defined in terms of the ecological context, the human benefit, and the connection(s) between them. Further complicating these descriptions is that the connections are often not linear, one-to-one relationships. A single environmental attribute may provide multiple benefits and a single benefit may rely upon multiple attributes. Therefore, a classification system provides an organizational framework to describe EGS consistently and comprehensively in efforts to assess, measure, quantify, map, model, or value the impacts of environmental changes. In general, classification systems offer the benefits of a unifying language, an understanding of how each category and its elements are related, improved identification of metrics, and improved knowledge transfer among research efforts (Finisdore et al., 2020).

Various ecosystem service classification systems have been developed to organize and clarify the wide-ranging kinds of ecosystem services, enabling discussions, (biophysical and economic) modeling, and welfare valuation. By making the link between ecosystem services and well-being explicit, decision makers have become more informed when implementing new policies (Ruckelshaus et al., 2015; Barton et al., 2018; Dunford et al., 2018). The most prominent classification systems^2^ to date include the MEA, The Economics of Ecosystems and Biodiversity (TEEB) project, the Common International Classification of Ecosystem Services (CICES), and the National Ecosystem Services Classification System Plus (NESCS Plus) (see Finisdore et al., 2020 for more detail on these and other classification systems).

The MEA began in 2001 as an ecological project under the United Nations Environmental Programme (UNEP). It was established to help develop the knowledge base for improved environmental policy decision-making (Duraiappah et al., 2005). Ecosystem services are categorized into provisioning (e.g., food, water, timber, fiber), regulating (e.g., services affecting climate, flooding, disease, waste, and water quality), supporting (e.g., soil formation, photosynthesis, nutrient cycling), and cultural (e.g., recreational, aesthetic, and spiritual benefits) (Duraiappah et al., 2005). The MEA is a conceptual model of the interactions between biodiversity, EGS, well-being, and human drivers of change (e.g., population growth, technological advances, and lifestyle changes). In addition, it accounts for the spatial and temporal dimensions that influence these interactions. The advantage of this conceptual model is that it incorporates the full complexity of ecosystem services and their interactions with humans to assess welfare impacts. However, the MEA has several disadvantages. First, including supporting (or intermediate) services, which provide indirect societal welfare benefits by maintaining processes necessary for the other types of ecosystem services, can lead to double counting the economic value of a service (Fisher et al., 2009)^3^. Second, it does not differentiate either the uses or users of a service. Third, its delineation of provisioning service mixes ecosystem activity together with human activity making accounting and analysis imprecise. Despite its issues, the MEA has done much to boost awareness that protecting ecosystems (and maintaining their functioning and deliverability of services) is necessary to preserve peoples’ well-being (Mulder et al., 2015). The classification systems described below, which attempt to address these issues, have all been inspired or influenced by the MEA.

The TEEB was initiated by researchers in Germany and the European Commission. Unlike the MEA, the TEEB was designed to improve decision makers’ understanding of the economic significance of ecosystem services and their provided natural capital (De Groot et al., 2010). The TEEB revised the MEA classification by removing the supporting services category (which is viewed as a subset of ecological processes) and including a new category, “habitat”, to highlight the importance of habitat provision. To clarify the links between ecosystem services and well-being, the TEEB connects ecosystem functions (which represent the potential or capacity for an ecosystem to deliver a service), ecosystem services, and benefits. This separation of ecosystem functions and ecosystem services was done to prevent double counting the economic value of benefits. Ecosystem services and benefits are also separated; ecosystem services are viewed as the contributions that ecosystems make to well-being as flows (e.g., a constant flowing stream), whereas ecosystem benefits are stocks created (from the combination of natural and non-natural capital) or derived by people from those services (e.g., hydroelectric power generated from a dam). Lastly, although economic benefits are the main focus of the TEEB, the system also separates benefits into ecological (i.e., valuing the integrity or health of one part of an ecosystem derived from another, such as the value of one species for the survival of another), and sociocultural (i.e., valuing biodiversity or ecosystems for their provided health, historical, ethical, religious, or spiritual significance) categories (De Groot et al., 2010).

The CICES was developed by the European Environment Agency to provide a science-based, hierarchical classification system for environmental accounting and to map the supply of ecosystem services. It was designed with the intention of supporting those interested in quantifying the value of ecosystem services, as well as those interested in assessing how human impacts alter an ecosystem’s capacity to deliver services (Haines-Young and Potschin, 2012). The CICES was the first classification system to use a hierarchical structure for classifying ecosystem services. This structure was chosen to provide flexibility in its applications across various thematic and spatial scales and includes all of the ecosystem services identified in the MEA except for the supporting services. The CICES distinguishes between final and intermediate ecosystem services, although a lack of explicit partitioning between final and intermediate services (despite excluding supporting services) may not prevent double counting (Haines-Young and Potschin, 2018; Newcomer-Johnson et al., 2020). Ecosystem services are explicitly indicated as final services that directly benefit people, while biophysical structure and function are intermediate (supporting) services. This was done to link ecosystem and economic accounts, which necessitated identifying those final services of value to people (Haines-Young and Potschin, 2012).

The National Ecosystem Services Classification System-Plus (NESCS Plus) and webtool were developed by the EPA (U.S. Environmental Protection Agency, 2015; Newcomer-Johnson et al., 2020). Like CICES, a hierarchical structure is used to classify ecosystem services; however, the NESCS Plus focuses on FEGS and takes a beneficiary perspective, linking biophysical attributes of ecosystems to specific benefits or uses for human stakeholders. The system uniquely identifies distinct categories of FEGS and the pathways through which they impact well-being, supporting quantitative analyses of benefits from ecosystem services. The NESCS Plus offers two ways to classify the human dimensions (i.e., the receiving end) of FES flows:

1. A combination of Direct Use and Direct User classes and subclasses; and
2. Beneficiary classes and subclasses.

Both approaches use hierarchical lists to support a comprehensive identification of the different ways in which humans benefit from ecosystems. Regardless of the approach selected, the user will receive results for Direct Use, Direct User, and Beneficiary classes. Using the combination of Direct Use and Direct User components provides the flexibility to separately classify: (1) how an ecological end-product or environmental attribute is used; and (2) who uses it. Following established classification structures adopted by the U.S. Census Bureau and United Nations, the first level includes broad sectors of the economy – Industry, Households, and Government. By using the North American Industrial Classification System (NAICS) system to classify who (i.e., identify the Direct User), it also offers an easy link to other information systems that use NAICS categories or codes to classify economic or other data as NAICS is the standard used by U.S. federal statistical agencies in classifying business establishments. The Beneficiary approach is simpler because it only contains one component, and thus it may be more intuitive, especially for users with less experience with NAICS. Unlike the Direct User and Direct Use/Non-Use concepts, the Beneficiary concept does not separate the questions of: (1) who benefits from nature; and (2) how they benefit. Therefore, it can be considered a combination of the two concepts.

## 3 NESCS Plus, common language, and decision-making

Although the NESCS Plus was designed to support systematic and comprehensive accounting of changes in FEGS, the primary motivation behind its development was to provide a robust, step-by-step resource for taking a human-centered approach to tracing the links between ecosystems and human well-being. The primary purpose of the NESCS Plus is to serve as a framework for analyzing how ecosystem changes impact human welfare. In the NESCS Plus, the EPA provides a means to standardize ecosystem services classification (e.g., Bell et al., 2017; Rhodes et al., 2017; Bolgrien et al., 2018; Littles et al., 2018; Angradi et al., 2019; Tashie and Ringold, 2019; Yee et al., 2019; Warnell et al., 2020; Jones Littles et al., 2023). This solid foundation can be used in the further use and development of ecosystem services research and in developing other ecosystem services tools that use the same “language”. While the NESCS Plus provides a framework, architecture, and taxonomy it is not a decision support system on its own. Through integration with other tools like the FEGS Scoping Tool it can support decision-making. The intended audience for this resource includes individuals, communities, private and public sector firms, and non-profit organizations looking to measure, quantify, map, model, and/or value a comprehensive standard set of ecosystem services anywhere on the Earth.

The NESCS Plus lists of (1) beneficiary classes and subclasses and (2) ecological end-product categories and environmental attribute subcategories (hereafter referred to as attributes) provide the foundation for consistent and comprehensive descriptions of human benefits and the underlying ecosystem using language that is clear and easily understood by a range of audiences. No additional translation is needed when using the NESCS Plus to describe a *Recreational Hunter* who cares about *Edible Fauna* in a *Forest* to the recreational hunter and therefore to scientists, decision makers, or the general public. This clarity is essential for ensuring that these different groups have a common understanding of the ecosystem and their connections to it. The systematic and comprehensive approach supported by the NESCS Plus is also helpful for evaluating tradeoffs between the benefits, a significant advantage since a single change to an ecosystem can impact different beneficiaries in a variety of ways. Finally, the hierarchical nature of the beneficiary and attribute lists supports use of the classification system at a variety of scales. The system does not specify or limit the spatial or temporal scale and allows the system user to specify these dimensions based on their own needs and context. For example, users could choose to use the broader top-levels of the hierarchy, or they could even create their own finer nested levels by providing additional specificity to descriptions of beneficiaries or attributes, users are able to describe FEGS at more local scales while maintaining the connection to and benefits of the NESCS Plus. To return to the above example, the FEGS can be described more precisely for decision-making purposes as *Recreational Bow Hunter* who cares about *White Tail Deer* in *Francis Marion National Forest* without losing the connection to the NESCS Plus standardized language and its associated benefits.

## 4 The EPA*’*s final ecosystem goods and services tools

In general, decision-making is a six-step process, regardless of whether the steps are considered explicitly (Gregory et al., 2012). Ecosystem services are a valuable inclusion at every decision step (Table 1) and researchers are working to support that (Yee et al., 2017; Fulford et al., 2023; Yee et al., 2023a). As decision makers tackle a range of decisions, with varying levels of resources, complexity, and public interest, the steps in which they include EGS will shift. To be as useful as possible to decision makers, researchers must support that flexibility with a flexible, scalable approach that can be consistently applied to increase usage, comparison, and transferability.

Researchers from the EPA are identifying and quantifying ways in which natural ecosystems contribute to healthy and sustainable communities (Harwell and Jackson, 2021). An explicit goal is to provide information and tools that help decision makers and local communities sustain such contributions, known as ecosystem services, to enhance aspects of human well-being, including economic growth and prosperity, public health, stability, and resiliency. Using the standardized framework and language of the NESCS Plus classification system allows tools and databases to interact, making it easier for decision makers to move from one to another and to combine tool use to best meet their needs for where they are in their decision process. A common language can allow tool inputs and outputs to intersect with each other and simplify communication of tool results. For decision makers, NESCS Plus addresses the question: “How do we start talking about ecosystem services?”

The EPA has four tools that can work with the NESCS Plus language: (1) the FEGS Scoping Tool; (2) the FEGS Metrics Report; (3) the EnviroAtlas; and (4) the EcoService Models Library. Each tool addresses different decision maker needs and can be used at different points in the decision-making process (Table 2). The FEGS Scoping Tool and the FEGS Metrics Report underwent development as the NESCS Plus was being finalized and each was designed to use the NESCS Plus beneficiary and attribute lists as critical structural elements. The EnviroAtlas and the EcoService Models Library were developed and released before the NESCS Plus and the common language was included subsequently. All four tools, described below, benefit from the coordinated implementation that the NESCS Plus makes possible. This creates a synergistic capacity for using multiple tools as part of a decision-making process.

### 4.1 The FEGS Scoping Tool

The Final Ecosystem Goods and Services (FEGS) Scoping Tool (https://www.epa.gov/eco-research/final-ecosystem-goods-and-services-fegs-scoping-tool) was developed to meet the needs of researchers and managers interested in identifying relevant EGS for a particular project, area, or decision context. It answers the question: “What ecosystem services matter?” Although this seems like a straightforward question, there are a few potential complications. First, the ecosystem under consideration will be producing, or capable of producing, a certain set of EGS. These EGS, however, may or may not be of interest. Therefore, identification of beneficiaries must be done to identify relevant FEGS. Second, ecosystems can produce a wide range of FEGS and beneficiaries are interested in a wide range of ecosystem services. The list of FEGS for any given area could be considerable and cannot all reasonably be considered or evaluated in decision-making. Extensive lists of potential items of interest are of limited utility in decision-making as they provide a wide range of options but no way of distinguishing among them (Scheibehenne et al., 2010). The FEGS Scoping Tool addresses these issues by providing users with a transparent, repeatable, and defendable approach for identifying and prioritizing the FEGS most relevant to a decision’s stakeholder groups (Sharpe et al., 2020).

The importance of including stakeholder perspectives in decision-making has long been recognized (Gregory, 2000; Gregory and Wellman, 2001), but constraints may limit the extent and scope of stakeholder involvement and necessitate some degree of prioritization for inclusion. If not done explicitly, this prioritization is often done in an unconscious or *ad hoc* fashion. The FEGS Scoping Tool takes a formal approach towards stakeholder prioritization and then uses the results of that prioritization to subsequently identify and prioritize the ways in which stakeholders are connected to the environment and the specific aspects of the environment necessary for those connections (Sharpe et al., 2020). It is designed to be used at an early stage of the decision-making process. By focusing on the most relevant FEGS, rather than those most discussed or most easily measured, decision makers increase the likelihood that the consideration of FEGS will be influential in the decision-making process. By beginning the analysis with a complete consideration of all possible stakeholder groups, decision makers increase the likelihood of finding common beneficial uses among the stakeholder groups and decrease the likelihood that valued FEGS will be overlooked in the decision-making process.

The FEGS Scoping Tool uses decision criteria designed to support stakeholder prioritization (Sharpe et al., 2021). In the first stage, tool users identify stakeholder groups and prioritize them using the provided criteria. In the second stage, the NESCS Plus beneficiary lists are used to identify the ways in which each stakeholder group benefits from the potentially impacted ecosystem. The prioritization from the first stage carries through to result in a prioritized set of beneficial uses. In the third stage, the NESCS Plus attribute lists are used to identify the critical ecosystem elements for realizing each beneficial use. Again, the prioritization from the previous stage is carried through, and the result is a prioritized set of environmental attributes.

The FEGS Scoping Tool’s use of the NESCS Plus language amplifies the consistency of the tool, allowing decision makers to use completed tool runs as a starting point when considering the same stakeholder groups for a new decision. This clarity, comprehensiveness, and consistency makes the FEGS Scoping Tool an effective tool for fully characterizing the FEGS relevant for a decision context and identifying the subset that should be used as decision objectives.

As an example, a multi-stakeholder driven revitalization effort at the East Mount Zion Superfund landfill site in York County, Pennsylvania focused on improving the social-ecological value of the ecosystem by creating a space for education, recreation, wildlife habitat, and enhanced biodiversity making the site an asset to the community. The team used the FEGS Scoping Tool to work through stakeholder priorities and identify desired EGS to target (Sharpe et al., 2022). In another example, the FEGS Scoping Tool was used to understand priorities of Chesapeake Bay stakeholder groups in connecting EGS – identified using NESCS Plus – and beneficiaries associated with Best Management Practices in the watershed (Rossi et al., 2022).

### 4.2 The FEGS Metrics Report

Interest in including EGS in decision-making has grown in recent years to more fully account for nature’s contributions to human and environmental health (Posner et al., 2016). The challenge for U.S. federal agencies has been that much of the EGS research has been at small spatial extents or specific case studies, making it more difficult to consider regional and national scales at which federal policy is made. The need for a consistent metrics for EGS assessment was identified by the EPA Science Advisory Board (U.S. Environmental Protection Agency, 2009), which found – despite large, nationwide ecological monitoring programs – that analysis of EGS was problematic due to a lack of specific metrics that can represent changes in ecosystem services (e.g., Chestnut and Mills, 2005), and a lack of specific stakeholders who may benefit from these services (Ringold et al., 2013; U.S. Environmental Protection Agency, 2020). In this context, the EPA co-developed a standardized process to identify metrics that allow decision makers to answer the question: “How to measure what matters?”

This process, arising from a collaboration between social and natural scientists, was formalized in 2020 with the publication of the EPA report *Metrics for national and regional assessment of aquatic, marine, and terrestrial final ecosystem goods and services* (U.S. Environmental Protection Agency, 2020). Because these metrics have joint validity with both natural and social scientists, they can be used for interdisciplinary analysis across disciplines. This report helped to operationalize these ideas by providing specific metrics that can be used by both natural and social scientists for analysis. These metrics serve as linking indicators between these different ways of knowing and analyzing ecosystem services (Boyd et al., 2016). This joint validity exists because FEGS serve as the end-products of ecological systems and the inputs into social systems. Further, the report focuses on metrics that measure specific, tangible biophysical features or qualities that are relevant for management and that are provided in units that require little to no technical explanation. Beyond linking different scientific disciplines, these metrics also facilitate communication to lay audiences involved, invested, or interested in changes to the ecosystem being measured.

The metric report lays out a standardized, five-step process that can be used to identify metrics (U.S. Environmental Protection Agency, 2020):

1. Recognize ecosystem boundaries from the perspective of natural scientists.
2. Specify beneficiaries who use, interact with, or enjoy the ecosystem services created by the ecosystem.
3. Identify the biophysical components of nature that link the ecosystem and the beneficiary’s interests.
4. Describe the metrics for each beneficiary/attribute combination (e.g., each FEGS).
5. Consider the data availability of the potential metrics.

The report itself also includes suggested metrics for seven ecosystem types (coral reefs; estuaries; lakes; rivers and streams; wetlands; agricultural lands; forests) and more than 40 different beneficiaries as a starting point and example of the process.

As with the FEGS Scoping Tool, the FEGS Metrics Report was built upon using the NESCS framework to help users specify the biophysical measures that were most relevant for a specific beneficiary/attribute combination. The Report benefits from the NESCS Plus framework being sufficiently comprehensive for encompassing all beneficiary/attribute combinations for which metrics may be needed as well as from its scalability, allowing users to craft metrics relevant to assessments ranging from the national to the local. Communicability, comprehensiveness, and scalability make the FEGS Metrics Report an effective tool for identifying decision objective measures that can be used when estimating consequences.

Although the report was not publicly available at the time of the work conducted in Chesapeake Bay, researchers used the same foundational framework as the report (Ringold et al., 2013) when identifying the metrics most relevant to priority FEGS. Those metrics assist managers in encourage adoption of Best Management Practices by connecting them to the beneficial uses valued by the community (Rossi et al., 2022).

### 4.3 EnviroAtlas

The EnviroAtlas (https://www.epa.gov/enviroatlas) allows users to visually interpret ecosystem services and understand how they can be included in decision-making efforts, answering the user question: “How to map what matters?” The EnviroAtlas was developed collaboratively between the EPA, the U.S. Geological Survey, the U.S. Department of Agriculture, and other federal and non-profit organizations, universities, and communities including state, county, and city-level stakeholders. The EnviroAtlas is an online mapping resource containing more than 500 geospatial data layers for the U.S., including environmental and socioeconomic related data and tools that can be used to examine a location and characterize the ecological and socio-economic status (Pickard et al., 2015). These data can be used in an EGS framework. Using an interactive, online map approach, the EnviroAtlas contains EGS data organized into several categories characterizing the production, demand, and the EGS attributes that may affect an ecosystem’s ability to produce EGS. The EnviroAtlas has seven overarching EGS benefit categories: food, fuel, and materials; clean air; recreation, culture, and aesthetics; natural hazard mitigation; climate stabilization; clean and plentiful water; and biodiversity and conservation.

The EnviroAtlas was designed for multiple audiences, (e.g., individual, government, or organization with an interest in the environment) and for use without special expertise. There are many potential applications of EnviroAtlas, including green infrastructure, brownfields, community planning, stormwater management, mitigation banking, and climate change resiliency, such as urban heat island abatement planning. The EnviroAtlas contains data at two primary scales. Many data layers are available at the national scale, with approximately 97,000 sub-watershed hydrologic unit codes (HUC 12) for the conterminous U.S. (ranging from 39 to 160 km^2^ each). Most of the community data layers are summarized at the U.S. census block scale. Additionally, 1-meter resolution land cover data exists for over 1,400 U.S. municipalities. The EnviroAtlas includes two climate change tools in an interactive mapping application. Additionally, the EnviroAtlas includes ecosystem markets data layers for market initiatives and enabling conditions at a range of scales. Although EviroAtlas was released prior to the NESCS Plus, the EnviroAtlas now includes a searchable matrix which crosswalks EnviroAtlas metrics with FEGS; again, providing a connection to the language of the NESCS Plus (Tashie and Ringold, 2019).

Bolgrien et al. (2018) used the EnviroAtlas to guide selection of EGS indicators, which were used as endpoints in a framework used to help stakeholders evaluate the community’s land use and infrastructure recommendations.

### 4.4 EcoService Models Library

In an ideal world, there would be ample time and money to measure all the EGS and FEGS that matter in every scenario. In the real world, time and money are often limited. After using the FEGS Scoping Tool and the FEGS Metric report to identify the FEGS and metrics that matter the most in a given scenario, it may not be feasible to measure them all so modeling may be a useful alternative. The EcoService Models Library (Bruins et al., 2017; DeWitt et al., 2020; Newcomer-Johnson, in press; https://www.epa.gov/eco-research/ecoservice-models-library) was developed to address the question of “How to model what matters?” by helping users find models to estimate the production of ecosystem goods and services.

The EcoService Models Library is a searchable database containing detailed descriptions of over 280 ecological models, their variables, and the source documents that describe them for use in estimating the production of ecosystem goods and services. Relationships potentially described as ecological models can vary widely in complexity, presentation, and subject matter. Some like InVEST and i-Tree are elaborate simulation tools with software, manuals, and websites (e.g., Nowak et al., 2013; Smith et al., 2017), while others are simple equations not found beyond the pages of a journal article (e.g., Bellinger et al., 2014), an ecological model can draw from a single discipline (e.g., a predator–prey interaction) or many (e.g., including physical–chemical–biological, and potentially social–political–economic elements). The EcoService Models Library was developed to help planners, analysts, risk assessors, economists, and other scientists to understand and select useful ecological models. A secondary purpose is to help researchers interested in improving ecological modeling methods.

Ecological models can be useful for linking ecosystems, stressors, and management actions to the production of EGS and FEGS. The ideal model for a particular issue should address the desired modeling objectives, should apply within the appropriate environmental context, would require the right degree of effort and expertise, and should characterize the level of uncertainty (Bruins et al., 2017). The EcoService Models Library’s 20 pre-defined filters are more powerful for most uses because they examine the EcoService Models Library classification-based descriptors; the pre-defined filters are source/collection, environmental sub-class, ecosystem services, hazardous waste site ERA, location, variable classification, time dependence, time continuity, spatial extent area, spatial distribution, computational approach, determinism, statistical estimation, calibration performed, goodness of fit reported, uncertainty analysis performed, ecological scale, and organismal scale. The filters can be used in combination to increase search specificity.

Most entries in the EcoService Models Library describe specific applications of models. Since model formulations often change from one application to the next, focusing on a specific application minimizes the problem posed by model versioning. Applications also include valuable information on context and often on uncertainty as well. Each model entry includes over 50 individual descriptors covering the model identity and description, modeling approach, location, environment, ecology, EGS potentially modeled by classification systems, and variable names. The environmental components (and language) of the NESCS Plus and the CICES are included. A variable relationship diagram, showing logical relationships between variables, is provided for each ecological model (Bolgrien et al., 2018). In addition to its main purpose of finding models, users can also find information about variable values used in model applications and examine the potential for linking models by sorting variables into Variable Classification Hierarchy top level categories. Each model variable is described by 40 additional descriptors. These variable descriptors are divided into variable general information, typology, spatial characteristics, temporal characteristics, values, variability and sensitivity, and operational validation.

**FIGURE 1:**
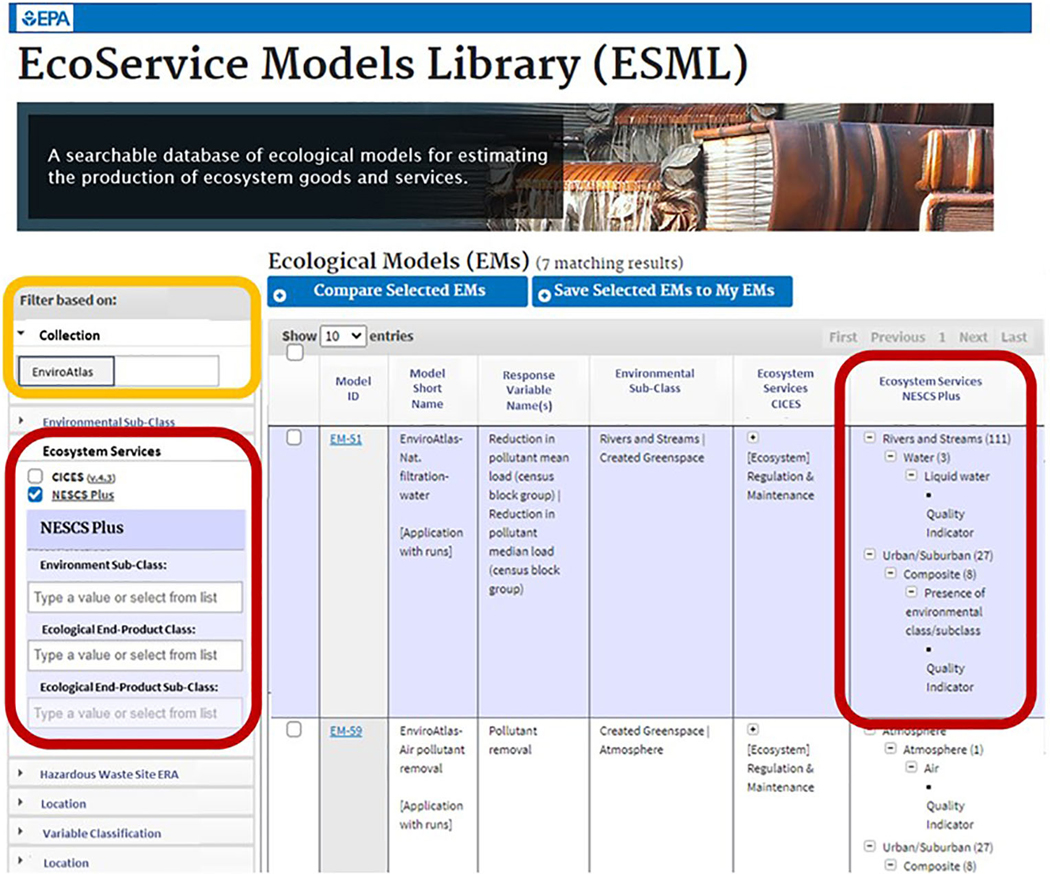
The EcoService Models Library (ESML) offers users the ability to find models using filters such as ecosystem services, using the NESCS Plus classes and subclasses (red boxes), as well as collections such as EnviroAtlas (yellow box).

In addition to links to the NESCS Plus classification system, the EcoService Models Library also has links to the EnviroAtlas (Bolgrien et al., 2018). For example, the EnviroAtlas is included as one of the collections that users can search for finding models (Figure 1). Additionally, an EnviroAtlas URL is included when data from the EnviroAtlas may be helpful for finding data to run the models found in the EcoService Models Library. The EcoService Models Library also includes links to EnviroAtlas fact sheets that provide information on how the data were created, limitations of the data, how to access the data, where to get more information, and references for selected publications. The EcoService Models Library matches ecological model variables to potentially useful EnviroAtlas data layers based on how the variables were classified in the Variable Classification Hierarchy, a classification system that bins variables into informative categories to enable searching and investigation of models based on their variable characteristics.

Returning to the East Mount Zion example, the revitalization team also used the EcoService Models Library to ultimately identify five ecological models (e.g., carbon storage and sequestration, pollinator populations, rare species, and bird populations) relevant to their targeted EGS to apply to examine scenarios. As a result, the team was able to examine a broader suite of EGS they might not have otherwise identified as target endpoints. The EcoService Models Library was also used in the Chesapeake Bay example to help identify potentially relevant metrics.

## 5 More powerful than the sum of their parts

We posit that using multiple tools with the common anchoring point of the NESCS Plus framework creates a synergistic effect, much like the synergistic effects realized by using multiple teaching tools in a hybrid learning environment (Cai and Wang, 2022) or the synergistic effects of the legendary *Voltron*: *Defender of the Universe*, a robot with greater strength and skill than the five individual robot lions that make it up (Keefe, 1984–1985). The East Mount Zion example above used two of the four tools. By coupling the application of the FEGS Scoping Tool with the EcoService Models Library, the team realized synergistic outcomes not realized elsewise. Using the FEGS Scoping Tool to capture the community’s relationship to the site supported straightforward and evidence-based selection of the models chosen from the Library to assess future site scenarios and consistent use of the NESCS Plus beneficiary and attribute categories reduced the need for explanations as the team moved from identifying EGS to prioritizing them and modeling them. For East Mount Zion, working with multiple tools was more powerful than the sum of their parts and it led to more informed alternatives for their decision-making process.

These tools are applicable to different parts of the decision-making process and are useful, alone or together, for managers attempting to make decisions or take actions with an environmental component. However, incorporating one new tool into management processes can be time-consuming and unappealing to managers who are often both resource and time-limited, let alone incorporating multiple new tools. How priorities are chosen depends on the user and the decision context. As a result, an ecosystem services assessment tool selection portal was developed to walk the user through which tools are relevant for different parts of a generic, structured decision-making process (Harwell et al., 2023).

Given the recency of some of the tools’ release, we do not yet have a published example of all four tools being used together in the field. We do, however, have multiple examples that demonstrate the value of the tools when used in combination. In East Mount Zion, the consistent language provided clear connections between stakeholder values and the ecosystem service models selected to evaluate site scenarios. In Chesapeake Bay, tool compatibility allowed researchers to use one tool (the FEGS Scoping Tool) to gather and organize information on community priorities and two other tools (the FEGS Metrics Report, the EcoService Models Library) to select metrics responsive to those priorities and associated with the management organization’s Best Management Practices. Although the FEGS Scoping Tool was not yet publicly available at the time of the Milwaukee case study, researchers flagged its utility for use in combination with the EnviroAtlas and how it could be used within their framework (Bolgrien et al., 2018). Finally, the FEGS Scoping Tool, the EcoService Models Library, and EnviroAtlas were selected for coordinated demonstrations at a workshop aimed at exploring the incorporation of EGS tools into the ecological risk assessment process for contaminated sites (Kim et al., 2023).

### 5.1 Synergistic example

The above examples support our contention that the consistent EGS framework of the NESCS Plus would allow for the easy integration of all four tools into a single decision context. To that end, we lay out a hypothetical example using a common scenario demonstrating how that might be done. Pensacola Beach, in the Florida Panhandle, is known for its ultra-white sand beaches and is located just south of Pensacola, the second largest city in the Panhandle. A popular tourist destination and local resource, the land is owned by the government and leased to private and public entities. Currently, 60% of the land is held for public use with the rest leased for residential or commercial uses. For the purposes of this example, we consider a scenario in which county commissioners are evaluating the proposed construction of a new beachfront hotel.

**FIGURE 2:**
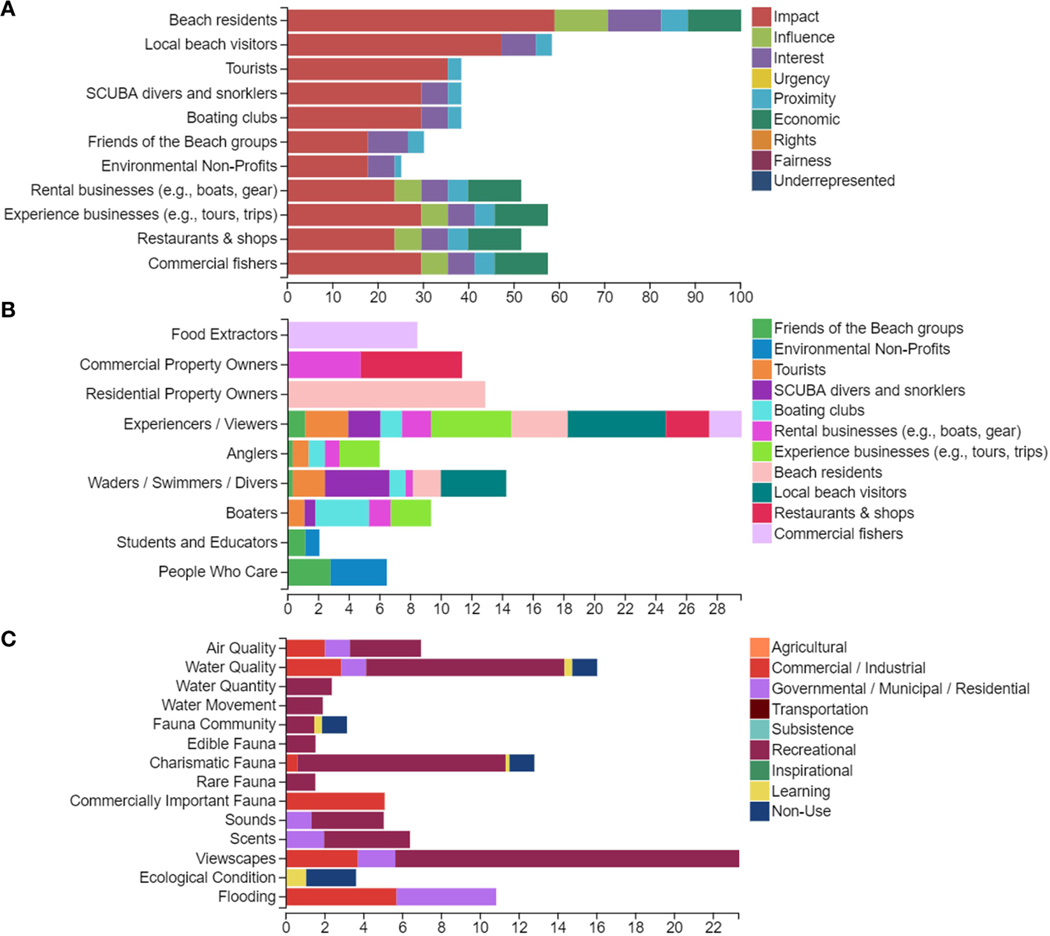
An example set of the FEGS Scoping Tool outputs. Panel **(A)** is a stakeholder prioritization (stage 1). It is the result of users weighting a set of decision criteria to reflect their values being used in this decision and scoring stakeholder groups on the extent to which they meet the criteria using tool-provided rubrics. These results arise from the hypothetical example in which commissioners are using the tool to prioritize stakeholder groups and the EGS they value when assessing the beachfront hotel development proposal. Here beach residents are a high-priority group because of the potential impact this decision would have on them. Panel **(B)** is a beneficiary group prioritization (stage 2) resulting from commissioners identifying the beneficiary groups making up each stakeholder group. Here we see beach residents benefiting as property owners, but also as experiencers/viewers and swimmers and divers. Panel **(C)** is an environmental attribute prioritization (stage 3) resulting from commissioners identifying the attributes needed for each beneficial use. For example, to enjoy their benefit, swimmers and divers need attractive viewscapes and charismatic fauna as well as water safe for immersion. The Scoping Tool outputs help commissioners identify the stakeholder groups (e.g., beach residents) whose perspectives should be included in decision-making and the ecosystem services that matter to them (e.g., the viewscapes from homes on the beach and the risk of flooding to those homes).

Commissioners might begin by selecting the criteria to use when evaluating the development proposal. There are several socioeconomic criteria that can be easily identified (e.g., jobs created, tax revenue, traffic, infrastructure needs). The FEGS Scoping Tool, anchored to the NESCS Plus, can help them identify EGS criteria that should be considered as well (Figure 2). In this example, commissioners review the tool’s list of stakeholder prioritization criteria and set “Magnitude and Probability of Impact” as the most important criterion for distinguishing among stakeholder groups. The FEGS Scoping Tool results indicate that those currently living or working on or near the beach (residents, rental and activity businesses, shops and restaurants, and commercial fishers) are the highest priority stakeholder groups (Figure 2A). Stakeholders benefit from the area in primarily recreational ways, and every stakeholder group benefits to some degree by being able to engage in experiencing/viewing activities. Wading, swimming, diving, and property ownership are also beneficial roles of high importance (Figure 2B). The results identify priority ecosystem services as viewscapes, water quality, and charismatic fauna for recreational uses and flooding related to commercial and residential uses (Figure 2C). This points the commissioners to including criteria related to these services alongside the socio-economic criteria when evaluating the proposal.

Once priority EGS have been identified, the commissioners need appropriate metrics to assess them. For some of the EGS, the FEGS Metrics Report can suggest metrics along with information about available datasets. Those metrics are organized by ecosystem type and identified using the same beneficiary and attributes lists as the FEGS Scoping Tool. For example, Table 3 contains metrics from report for viewscapes, water quality, and charismatic fauna for a range of recreational uses. For those criteria which do not have a corresponding metric in the report or for those where the suggested metric is inappropriate, the report contains guidance for developing more using the same format. In those cases, the results from the FEGS Scoping Tool can be used to complete the first three steps in the metrics identification process.

After the commissioners have identified and decided how to measure changes to priority EGS, they may be interested in assessing how those services may be impacted under different development scenarios. To do this, they can turn to the EcoService Models Library. Using the EcoService Models Library filtering system, they will easily be able to target models relevant to their priority services and metrics. For example, using the Ecosystem Service filter, they can find 12 models relevant to “Scapes: views, sounds and scents of land, sea, sky, or a combination.” By reviewing the models’ response variables, the commissioners can easily find the set of models most helpful in evaluating development scenarios (Table 4).

**FIGURE 3:**
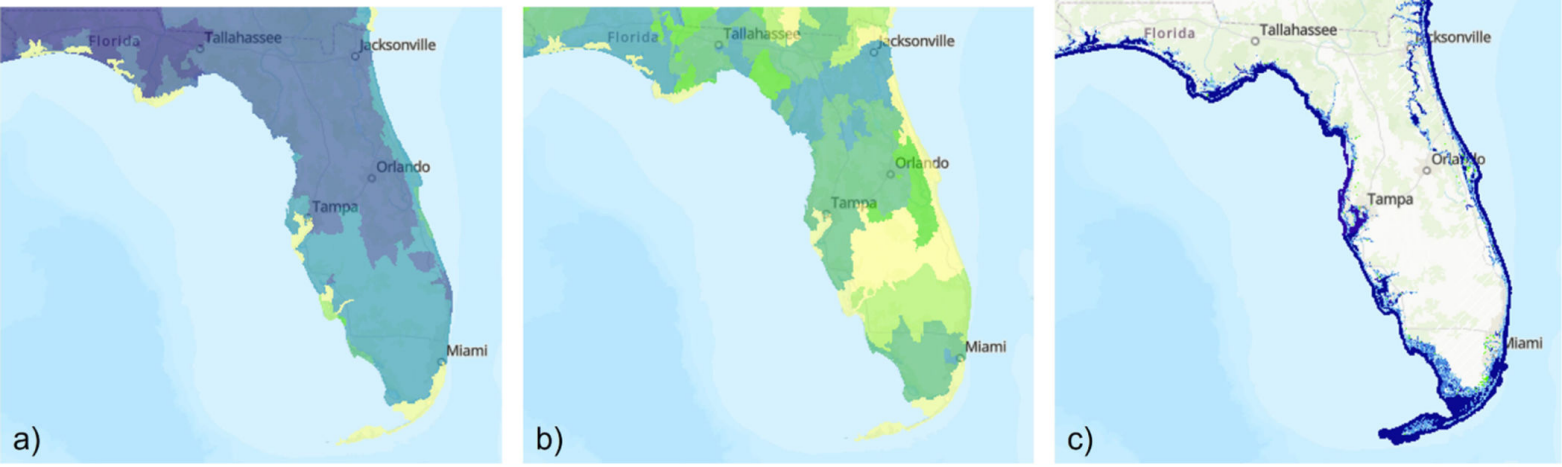
The EnviroAtlas data layers related to species richness **(A)**, rarity **(B)**, and sea level rise **(C)**. In the first and second parts of our hypothetical example, the FEGS Scoping Tool was used to identify the priority ecosystem services, including charismatic fauna for recreational uses and flooding for residential and commercial uses, and the FEGS Metrics Report was used to identify potential metrics for measuring recreational services, including species diversity and presence. Commissioners can search the EnviroAtlas to find data layers relevant to those priority services and metrics. This figure shows a subset of the data layers relevant to these services and metrics.

In addition to potential changes related to the production of EGS, commissioners are likely interested in the spatial distribution of priority services. Use of the FEGS Scoping Tool allowed the commissioners to identify charismatic fauna for recreational uses as a priority service and the FEGS Metrics Report suggested to measures of species diversity and presence. Now, the EnviroAtlas allows them to visualize data related to species richness and rarity, aspects of charismatic fauna that matter to a range of recreational beneficiaries (Figures 3A, B). The FEGS Scoping Tool also identified flooding for residential and commercial uses as a priority EGS and the commissioners can use the EnviroAtlas to find maps of flooding and sea-level rise (Figure 3C). The EnviroAtlas can also allow commissioners to visualize aspects of flooding related to the costs and viability of property ownership and to compare the suitability of different sites for the proposed development activity if 1-m resolution data are available.

Together, these tools allow the commissioners to identify the EGS of greatest concern to their stakeholder groups, determine how best to measure changes to those services in terms that are easily understood by a wide variety of audiences, and discover useful resources for exploring current and future levels of service production and spatial distribution. The tool ensemble allows for comprehensive consideration of EGS such that EGS criteria can be included in decision-making alongside the socio-economic criteria many decision makers are more familiar using. With this set of tools, the commissioners can feel justified in their decision of which EGS to include and how they are being evaluated.

## 6 Discussion

In our hypothetical Florida Panhandle example, we see that together, all four of these tools can work synergistically to allow the commissioners to more comprehensively consider how the EGS and uses valued by their constituents could be impacted by the proposed development than through the use of just one tool and allow for smoother integration than tools with different descriptions of EGS would permit. The FEGS Scoping Tool helps them focus on a finite list of priority ecosystem services and points them towards selection of metrics using the FEGS Metric Report process. The prioritized attributes can be used as search terms within the EcoService Models Library and the EnviroAtlas to facilitate identification of spatial data and models for estimating potential future changes. At every step throughout the process, the commissioners have a clear and communicable rationale as to which services they are focusing on and how those priority services pointed to the data used to make the decision. This consistency means that once the original EGS selection is complete, questions of which models and data to use are also answered. By anchoring these tools with the NESCS Plus language, the commissioners also have a consistent set of clear terminology they can use as part of their strategic communication efforts.

In addition to on-going work to utilize three or more of these tools under different decision context examples, we are exploring the next suite of EGS and FEGS tools using the NESCS Plus framework and language to set the stage for additional tools to ultimately connect to these existing tools. For example, the document analysis R code used in Yee et al. (2019), Yee et al. (2023a) and Jackson et al. (in press) focuses on development and application of an automatic document reader looking for FEGS word triplets (i.e., groupings of words capturing environment type, beneficiary/user, and environmental attribute). This too is anchored in the language of the NESCS Plus. As another example, from a social science perspective, researchers are compiling a large database of EGS case studies that includes information on a large suite of parameters – including language from the NESCS Plus – to allow for further analysis of governance (Yee et al., 2023b). The NESCS Plus has also recently been used to facilitate natural capital accounting for developing supply and use tables, to give a more complete picture of a local area’s environmental-economic trends (Warnell et al., 2020). Finally, as mentioned above, researchers have also recently developed an EGS assessment tool selection portal (Harwell et al., 2023) allowing the user to better understand what tool might be relevant for a given type of decision-making need (sensu Table 2). All of these efforts are focused on the core principles of having anchoring language to the NESCS Plus and are aimed at creating opportunities for increasing the synergistic powers of multiple tools being used at the same time. As different case study applications proceed, researchers will continue to refine our understanding of different ways these tools can be applied and develop ideas for enhancing tools or create new ones. The larger scientific community is invited to help us along this journey to facilitate further use of FEGS into decision-making. These tools are publicly available and can be used off-the-shelf to assist researchers and practitioners in better integrating ecosystem services into decision-making processes, and thus improving human and community well-being.

## Figures and Tables

**TABLE 1:** Ecosystem services for each step in a decision process (modified from Yee et al., 2017 and Harwell et al., 2023).

Decision-making step	Why EGS consideration can bring value
Clarify decision context	Helps clarify the decision’s potential impacts on stakeholders and the spatial and temporal extent of the impacts. Example activities: • Identify and prioritize stakeholders and EGS • Identify potential EGS using clearly defined terms and a comprehensive list • Find strategies for identifying relevant EGS objectives and impacts
Define objectives	Helps to identify measures related to stakeholders’ relationships with the ecosystem and identify the extent to which non-EGS objectives rely upon underlying EGS. Example activities: • Identify established links between EGS and human health • Identify most relevant and meaningful FEGS metrics • Identify potential EGS using clearly defined terms and a comprehensive list • Identify and prioritize stakeholders and EGS • Find strategies for identifying relevant EGS objectives and impacts
Develop alternatives	Supports identification of creative alternatives arising from an understanding of the connections between the ecosystem and human well-being. Example activities: • Identify potential EGS using clearly defined terms and a comprehensive list
Estimate consequences	Assesses impact of the alternatives to valued EGS objectives to reduce the likelihood of unintended consequences. Example activities: • Identify established links between EGS and human health • Map people and built spaces • Find models for estimating EGS • Create conceptual model for how stressors impact EGS • Estimate stressors and impacts on EGS • Map alternative land-use scenarios and EGS, and impacts • Examine EGS risks and benefits to compare and communicate decision alternatives
Evaluate tradeoffs and select	Allows for consideration of how different options meet EGS objectives alongside other social or economic objectives. Example activities: • Find strategies for evaluating EGS • Identify and prioritize stakeholders and EGS
Implement, monitor, and review	Helps evaluate the changes on the site to measurable changes in realized benefits. Example activities: • Identify most relevant and meaningful FEGS metrics • Identify potential EGS using clearly defined terms and a comprehensive list • Find strategies for incorporating EGS into monitoring

**TABLE 2:** The tools discussed in this article and the generic decision-making steps at which they are potentially useful.

Decision-making step	Tools
Clarify decision context	NESCS Plus, FEGS Scoping Tool
Define objectives	NESCS Plus, FEGS Scoping Tool, FEGS Metrics Report
Estimate consequences	EcoService Models Library, EnviroAtlas
Evaluate tradeoffs and select	FEGS Scoping Tool
Implement, monitor, and review	NESCS Plus, FEGS Scoping Tool

**TABLE 3:** Example FEGS metrics for viewscapes, water quality, and charismatic fauna for a range of recreational uses (adapted from U.S. Environmental Protection Agency, 2020).

Beneficiary			Attribute		Biophysical metrics		Datasets				
Beneficiary	Specific beneficiary	What matters directly to this beneficiary?	Subcategory	Specific attribute	Ideal	Currently available	Data source	Scale (national, regional, local) Extent	Temporal dimensions	Currently described over large areas via remote sensing? (yes/no)	Existing capacity to model over large extents? (yes/no)
Anglers	Catch & Release	Is this area aesthetically enjoyable?	Site Appeal	Scents & Viewscapes	Color of water, algae, clarity & smell, lack of sound	Local reports	Online Posting	Local	Seasonal	No	No
Boaters	Kayakers, SUPs, and Boaters	Is this reef aesthetically enjoyable?	Site Appeal	Scents & Viewscapes	Water clarity	Field crew opinion	Word of mouth, local bait & tackle shops, local radio & TV fish reports	NA	Daily	No	No
Waders, Swimmers, and Divers	Scuba Divers and Snorkelers	Is there interesting enough viewscape to entertain divers?	Site Appeal	Viewscapes	Colors, shapes, diversity, movement	Local reports	Online posting	NA	–	No	No
Anglers	Catch & Release	Is WQ sufficient to be safe for angling?	Water Quality	Chemicals & Contaminants	Personal contact with contaminants from water	Contaminant concentrations in water	NARS; EMAP	National, Regional	Single sample during baseflow	No	No
Boaters	Kayakers, SUPs, and Boaters	Is the water in the wetland safe for recreational boating?	Water Quality	Contaminant from water exposure (chemical & biological)	Individual wetland contamination metrics	Levels of harmful bacteria; levels of chemical contamination	2011 NWCA; EPA sources	National	–	No	No
Waders, Swimmers, and Divers	Scuba Divers and Snorkelers	Is there sufficient visibility to be pleasurable to divers?	Water Quality	Water Clarity	Secchi disk	Diver recorded visibility	Online posting: diver recorded visibility; NOAA: satellite	Local	Daily	No	No
Boaters	Kayakers, SUPs, and Boaters	Will I see what im expecting or any interesting animals?	Charismatic Fauna	Taxa & Presence	Species, size, abundance, diversity	Species, size, abundance, diversity	U.S. FWLS, NOAA, State fisheries departments (FWC)	Local Observations Aggregated to States Regions and the Nation	Seasonal	No	Yes
Waders, Swimmers, and Divers	Scuba Divers and Snorkelers	Do these species attract the beneficiary?	Charismatic Fauna	Taxa & Presence	Large marine organisms	Presence/absence	EPA, NOAA, State	Regional	Seasonal	Depends–species	Yes
Anglers	Catch & Release	Will I catch something interesting?	Charismatic Fauna	Taxa & Presence	Species, size, abundance, diversity	Presence/absence	State, Federal	Local Observations Aggregated to States Regions and the Nation	Seasonal	No	Yes

In the first part of our hypothetical example, the FEGS Scoping Tool was used to identify the priority ecosystem services, including viewscapes, water quality, and charismatic fauna for recreational uses. Once identified, commissioners can use the FEGS Metrics Report to find example metrics or to find the process for developing additional metrics. This table contains a subset of the metrics found in the report that the commissioners could use when assessing the development’s potential impact to priority recreational ecosystem services. NA, Not applicable.

**TABLE 4:** A subset of the results from an EcoService Models Library search for models related to viewscapes, soundscapes, and scentscapes.

EM ID	EM-193	EM-713	EM-419	EM-683	EM-686
Model Name	Cultural ecosystem services, Bilbao, Spain	ESII (Ecosystem Services Identification and Inventory) Tool, MI, USA	ARIES (Artificial Intelligence for Ecosystem Services) Scenic viewsheds for homeowners, WA, USA	Value of recreational use of an estuary, Cape Cod, MA, USA	Estuary recreational use, Cape Cod, MA, USA
Response Variables	Aesthetic quality of the landscape Recreation provision index	Aesthetics: noise (noise attenuation) Aesthetics: visual (visual screening)	Enjoyed views (actual source of scenic viewsheds for homeowners) Potential views (theoretical source of scenic viewsheds for homeowners) Ratio of actual to theoretical source of scenic viewsheds for homeowners	Estimated daily beach visitation Estimated daily visitation using landings and way to water/site Estimated daily visitors using boats Total estimated daily visitation	Percent daily boating use Percent daily kayaking/stand-up paddleboarding or rowing use Percent daily spending time by the shore use Percent daily walking use

Clicking the EM-ID number will take you to that model description in the online library. In the first and second parts of our hypothetical example, the FEGS Scoping Tool was used to identify the priority ecosystem services, including viewscapes for recreational uses, and the FEGS Metrics Report was used to identify potential metrics for measuring those services. In a third step, commissioners can use the EcoService Models Library to find models relevant to those services. This table contains a subset of the models found in the Library that the commissioners could use to predict the development’s potential impact to viewscapes.

## Footnotes

1 Although formally goods and services are distinct, we follow the common convention and use “services” to refer to both.

2 CICES and NESCS (Plus) are regarded as the only true classification systems, including categories that are complete, mutually exclusive, consistent, and relevant (Finisdore et al., 2020).

3 Fisher et al. (2009) describe intermediate ecosystem services as services which stem from interactions between ecosystem structure and processes. Intermediate services ultimately lead to final services, which in combination with other forms of capital provide human welfare benefits. For example, the intermediate service of nutrient cycling leads to the final service of clean water, which can be used for drinking or irrigation. The values of intermediate services are embedded in these welfare benefits, which is why their inclusion leads to double counting.
